# Zinc Oxide/Ag Water-Gated Field-Effect Transistor Coupled with Surface-Enhanced Raman Scattering for Indigo Dye Detection

**DOI:** 10.1021/acsomega.6c04192

**Published:** 2026-09-11

**Authors:** Rogério Miranda Morais, Theodoros Serghiou, Neri Alves, Carlos José Leopoldo Constantino, Jeff Kettle

**Affiliations:** † James Watt School of Engineering, 150833 University of Glasgow, Glasgow, Scotland G12 8QQ, U.K.; ‡ Department of Physics, School of Technology and Applied Sciences, 28108 São Paulo State University UNESP, Presidente Prudente, SP 19060-900, Brazil

## Abstract

Water-gated field-effect transistors (WGFETs) based on a solution-processed ZnO–Ag composite are developed as a complementary electrical–spectroscopic sensing platform, combining electrical detection of indigo in aqueous media with SERS-based molecular identification on the same ZnO–Ag active layer. The resulting WGFETs operate below 1.5 V and exhibit stable transfer characteristics with reduced gate leakage and reproducible hysteresis behavior. Threshold voltage shifts (Δ*V*_th0_) induced by indigo exposure were described using a Langmuir–Freundlich model, providing a phenomenological representation of the nonlinear concentration-dependent electrical response, rather than definitive proof of adsorption heterogeneity. An apparent electrical LoD in the nanomolar range was estimated using the 3σ criterion. Simultaneously, surface-enhanced Raman scattering (SERS) performed on the same active layer enabled molecular fingerprint identification with a signal-to-noise ratio >3 at 10^–8^ mol L^–1^. The integration of WGFET and SERS is presented as a complementary sensing strategy in which the WGFET provides a sensitive electrical response in water and SERS supplies molecular confirmation through the characteristic vibrational bands of indigo. These results demonstrate that hybrid ZnO–Ag interfaces provide a multifunctional platform for low-voltage electrolyte-gated electronic materials capable of sensitive dye pollutant monitoring, with molecular selectivity supported by spectroscopic validation.

## Graphical abstract

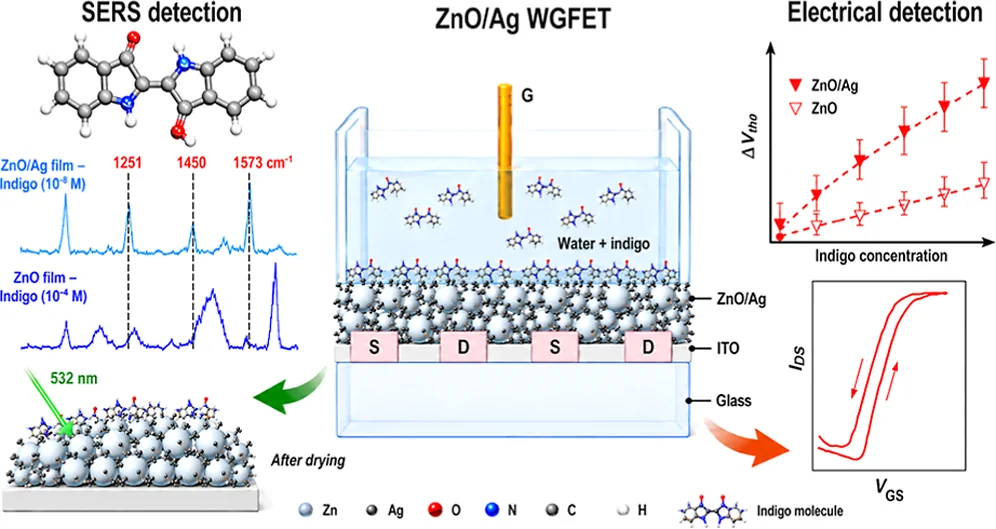

## 1 Introduction

Industrial-scale manufacturing has led to the intensive use of chemical compounds, which have inadvertently increased water pollution, making the monitoring of contaminants essential for environmental preservation and public health. According to the World Health Organization (WHO), more than 80% of global wastewater is discharged without proper treatment, contaminating the aquatic ecosystems. Among the most relevant global pollutants are natural and synthetic dyes, widely used in the textile, food, cosmetic, and printing industries.^1^

Indigo is one of the leading dye pollutants due to its large-scale use in the textile industry, with an annual consumption of approximately 40 000 tons, generating substantial chemical waste.^1^ Its low biodegradability and toxicity pose significant environmental risks, including water contamination and bioaccumulation, making the detection of indigo at low concentrations (<10^–7^ mol L^–1^) critical for pollution control and mitigation strategies.^2^ In natural waters, indigo exists in different chemical states depending on pH and redox conditions; under neutral to slightly alkaline conditions typical of rivers and lakes, base indigo predominates as a neutral, water-insoluble species that tends to aggregate or adsorb onto mineral and organic surfaces. Although this low solubility complicates detection by conventional analytical methods that rely on dissolved species, surface-enhanced Raman scattering (SERS) is a vibrational spectroscopic technique based on the enhancement of Raman scattering near-nanostructured surfaces. Here, SERS is used as a complementary molecular identification method to confirm indigo through its characteristic vibrational bands, allowing the identification of persistent indigo contamination in aquatic environments.^3^

Traditional detection methods offer high accuracy but are expensive, require complex laboratory infrastructure, and are not feasible for continuous monitoring in the field or point-of-use (POU) sensing.^4,5^ In this context, low-cost, portable, and sustainable electronic sensors represent a promising alternative. Among them, electrolyte-gated field-effect transistors (EGFETs) provide high sensitivity, operation at low voltages, and the ability to detect specific chemical variations in the electrolyte through the formation of the electric double layer (EDL) or changes in the surface potential of the semiconductor.^6^ Changes in the composition of the solution used as electrolyte, such as the presence of dye molecules, directly modulate electrical parameters such as the threshold voltage (*V*_th_), transconductance (*g*_m_), and drain-to-source current (*I*_DS_)_,_ allowing their application as real-time sensing platforms.^7–9^

Zinc oxide (ZnO) is an n-type semiconductor with a wide band gap (∼3.3 eV) and high exciton binding energy (∼60 meV), widely used in thin-film transistor, electronic, optoelectronic, and neuromorphic applications.^10–14^ Its high surface reactivity enables sensitive detection of gases, ions, and organic molecules, including organic dyes, through adsorption-induced charge modulation.^15,16^ ZnO is also a low-cost, non-toxic, and earth-abundant material with chemical stability, biocompatibility, and controlled aqueous dissolution, supporting transient electronics.^17,18^ While spray-coated ZnO nanoparticle films provide a simple and low-cost deposition route, their use in electrolyte-gated field-effect transistors for sensing remains limited.^19^ This work addresses this gap by introducing a ZnO-based EGFET with nanoparticle-engineered interfaces to enable highly sensitive, low-voltage operation.

From a manufacturing standpoint, spray coating offers a low-energy, solution-based, and vacuum-free deposition method compatible with large-area processing and benign solvents, significantly reducing fabrication complexity and environmental impact and enabling low-cost, disposable, and environmentally responsible electronics.^20^ An underexplored approach is the integration of ZnO with Ag nanoparticles for combined FET-based and surface-enhanced Raman scattering (SERS) applications. When incorporated as nanoparticles or ultrathin layers, Ag provides significant functional enhancement even at low concentrations relative to the host matrix, while preserving adsorption and sustaining localized surface plasmon resonances (LSPR) to enhance the Raman signal. In FET-based devices, Ag improves surface conductivity and charge transfer in the ZnO channel, leading to faster and more sensitive responses to molecular adsorption.^21^ Previous studies report enhancement factors (EF) on the order of 10^6^ and detection limits down to ∼10^–9^ M for target molecules in ZnO–Ag-based SERS platforms.^22^ The ZnO–Ag composite therefore combines the intrinsic sensitivity of a semiconductor with the amplified surface response of a plasmonic metal, enabling multifunctional electronic and optical sensing within a single material platform.

Recent advances in wastewater monitoring and remediation have explored microbial nanobiotechnology as a sustainable strategy for pollutant removal and wastewater treatment.^23^ In parallel, nano-optical biosensing based on complex light–matter interactions has expanded the possibilities for sensitive, miniaturized, and point-of-care optical detection platforms.^24^ These platforms include surface plasmon resonance (SPR), localized surface plasmon resonance (LSPR), surface-enhanced Raman scattering (SERS), fluorescence-based nanosensors, colorimetric assays, and photonic sensing systems, which can provide rapid response, high sensitivity, and molecular or spectral selectivity depending on the sensing architecture. In this context, SERS-based sensors have recently been explored for the detection of pollutants in water treatment, highlighting the relevance of plasmonic nanostructures for trace-level identification of contaminants through enhanced vibrational fingerprints.^25^ SPR-based multilayer optical sensors have been specifically investigated for wastewater monitoring applications.^26^ Other approaches have focused on photocatalytic nanomaterials, such as complex silver sulfide nanosystems, for wastewater remediation and clean energy production,^27^ as well as bacteriogenic metallic and semiconducting nanosystems as sustainable solutions for environmental and One Health-related challenges.^28^

Despite these advances, a remaining research and technological gap concerns the development of simple, low-cost, and integrated sensing platforms capable of combining real-time electrical transduction in aqueous media with molecular-level confirmation of the target analytes. Conventional analytical methods, such as chromatography, mass spectrometry, and laboratory-based optical spectroscopy, provide high analytical performance but often require expensive instrumentation, sample preparation, and centralized analysis. In this context, the WGFET/SERS strategy proposed here is not intended to replace established methods but to provide a complementary platform for local screening and integrated sensing, where the WGFET enables sensitive electrical monitoring in water and SERS provides molecular confirmation through the characteristic vibrational bands of indigo.

This work presents a multifunctional and environmentally oriented platform based on ZnO and ZnO–Ag films fabricated via green, solution-processed routes, enabling complementary electrical detection of base indigo using WGFET and optical identification through SERS. Complementary sensing enhances reliability, selectivity, and information content by correlating independent transduction mechanisms within a single sensing platform. WGFETs incorporating AgNPs exhibit reduced leakage currents and a more reproducible concentration-dependent *V*_th_ response. These results suggest that Ag incorporation modifies the ZnO/electrolyte interface, increasing its sensitivity to indigo exposure under the tested conditions, without implying definitive evidence of improved active-site homogeneity or a specific adsorption mechanism. Raman measurements further reveal a pronounced SERS response, enabling molecular identification of indigo down to 10^–8^ M, while the WGFET response provided an apparent electrical LoD in the nanomolar range under the tested conditions. Collectively, these results demonstrate the potential of ZnO–Ag nanocomposite films as a low-cost, multifunctional, and sustainable platform for environmentally responsible chemical monitoring.

## 2 Methodology

### 2.1 Materials

For this work, zinc oxide nanoparticles (≤40 nm average particle size, 20 wt % in H_2_O), ethyl cellulose (EC, 49.5% w/w), Ag nanoparticles (nanopowder, <100 nm particle size, coated with PVP dispersant, ∼0.2 wt %), and synthetic indigo dye (dye content 95%) were used. All materials were purchased from Sigma-Aldrich.

### 2.2 Zinc Oxide-Based Inks

In this work, 1 mL of the commercial ZnO dispersion was redispersed in 6 mL of a water/ethanol solution (50:50% by volume). An ethyl cellulose (EC) solution in ethanol (2 mg/mL) was added to the ZnO-NP redispersion and maintained under magnetic stirring for 2 h at 80 °C. Using a similar approach, Ag-NPs in powder form were added to the dispersion. Two distinct inks were obtained: ZnO-NP and ZnO–Ag inks. Table 1 presents the mass and volume of the solutions used for film formation.

**Table 1:**
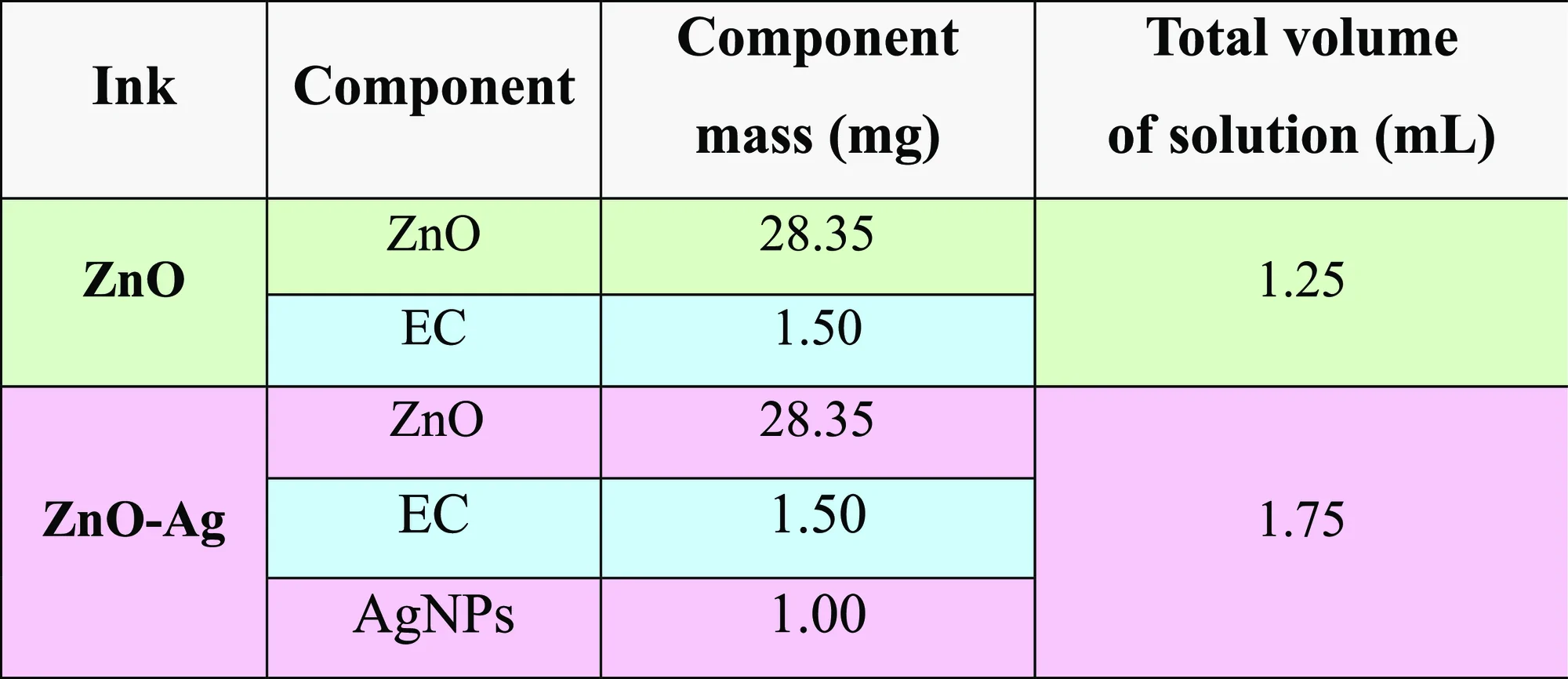
Composition of the ZnO-Based Inks

### 2.3 Indigo Dispersions

For testing purposes, the indigo dye was dispersed in ultrapure water (Milli-Q). The concentration of the dye was varied between 10^–8^ and 10^–4^ M. The dispersions were kept in a dark room and ultrasonically stirred for 30 min before being used. Figure S1 presents a photograph of the dispersions after ultrasonic agitation.

The sensing experiments were performed in ultrapure water to evaluate the initial response of the WGFET/SERS platform under the controlled conditions. However, this medium does not reproduce the complexity of real environmental waters, where salts, dissolved organic matter, surfactants, pH variations, and competing species may affect indigo aggregation, electrical double-layer formation, competitive adsorption, and the resulting WGFET response. Therefore, the present results should be regarded as a proof of concept rather than a complete validation of complex environmental matrices. In this context, the integration with SERS provides an additional molecular confirmation step since the characteristic vibrational bands of indigo can help distinguish the analyte from generic electrolyte-induced perturbations.

### 2.4 Semiconductor Layer Spray Coating

The devices were fabricated using commercially available pre-patterned ITO interdigitated electrodes on glass substrates purchased from Ossila Ltd., UK (S161-20). The substrates measured 20 × 15 mm and consisted of a 100 nm fully oxidized ITO layer with a nominal sheet resistance of 20 Ω sq^–1^. The transistor channels had a width of 30 mm and a length of 50 μm. The substrates were washed with acetone, dried under a stream of dry air, and treated for 2 min using an Ossila UV Ozone Cleaner equipped with four low-pressure mercury lamps emitting at 185 and 254 nm, with an irradiance of approximately 15 mW cm^–2^ at 185 nm. Subsequently, films based on ZnO-NPs were deposited on the substrates via an in-house spray-coating system. The system pressure was set at 1 bar, and the distance between the airbrush and the substrate was kept at 13 cm. To ensure that the same mass concentration reported in Table 1 was maintained in the dried films, volumes of 1.25 mL and 1.75 mL were used for the ZnO and ZnO–Ag inks, respectively. For film deposition, the inks were sprayed onto the IDEs heated to 150 °C, yielding films hereafter referred to as ZnO and ZnO–Ag. After the deposition process, the films were annealed at 150 °C for 1 h for the evaporation of solvents. Subsequently, the film surfaces were treated for 5 min using the same UV–ozone cleaner. The treatment was applied to promote the photo-oxidative removal of organic residues that could hinder analyte adsorption. These organic residues mainly originate from the solvent used in the ink formulation (ethanol) and from dispersing agents such as poly­(vinylpyrrolidone) (PVP) used to stabilize ZnO and Ag nanoparticles. UV treatment has been shown to effectively remove organic surface residues from nanoparticle surfaces by photo-oxidative cleaning, improving access of the analyte to the active surface sites^11,29^

### 2.5 WGFET Fabrication

The device was fabricated in the top-gate/bottom-contact architecture. For the electrolyte layer, 100 μL of ultrapure water was dripped into a polypropylene o-ring located on the surface of the films in the region of the IDEs. A gold wire, 1.00 mm in diameter, was inserted vertically into the volume of water and used as a gate electrode. The distance between the gate electrode, and the surface of the semiconductor films was maintained at 0.5 mm. The concentrations of indigo in water applied as the electrolyte were changed from the lowest concentration (10^–8^ M) to the highest (10^–4^ M). Figure 1a illustrates the production steps, while Figure 1b illustrates the structure, and Figure 1c shows a photographic image of the prepared devices.

**Figure 1:**
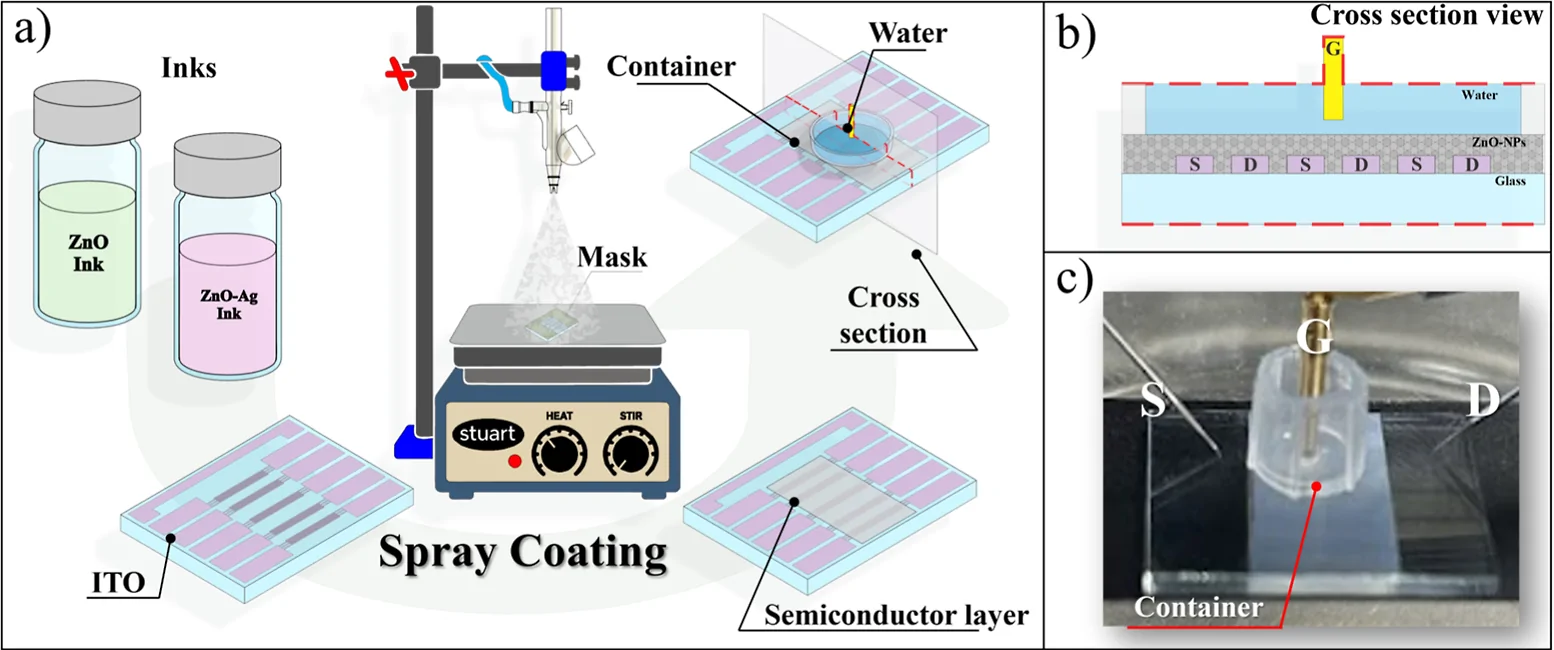
(a) Schematic illustration of the device fabrication process using spray-coating deposition. (b) Cross-sectional view of the WGFET architecture, highlighting the ZnO-based semiconductor layer, water electrolyte, and gate electrode. (c) Photograph of the device during electrical measurements, showing the positioning of the water reservoir and the source (*S*), drain (*D*), and gate (*G*) electrodes.

### 2.6 Test Equipment

Electrical characterizations were carried out using a Keithley 2636B model. For each device, six cycles of measurements were performed, keeping the potential between source and drain (*V*_DS_) constant at 0.5 V and varying the potential between the gate and the source (*V*_GS_) between 0 and 1.5 V in the dual sweep mode at a scan rate (d*V/*d*t*) of 50 mV/s. Raman spectra were acquired using a Horiba Jobin Yvon LabRAM HR Raman microscope with a 532 nm excitation laser line. Measurements were performed with a 50× LWD objective, 10 s acquisition time, and 10 accumulations. The laser was operated at a nominal output power of 1 mW, measured before losses along the microscope optical path and through the objective. After completion of the electrical measurements at each indigo concentration, the same devices were maintained under ambient conditions for 24 h, allowing the 100 μL volume of indigo-containing electrolyte used in the WGFET measurements to dry completely on the ZnO or ZnO–Ag active layer. Raman/SERS spectra were then acquired from the resulting dried residue in the same sensing region.

Although the Raman contribution from water is generally weak under SERS conditions, measurements in the aqueous phase exhibited additional background, optical scattering, and temporal instability. More importantly, base indigo has low solubility at near-neutral pH and forms a heterogeneous dispersion containing suspended particles and aggregates rather than a homogeneous molecular solution. The motion, sedimentation, and redistribution of these aggregates within the 100 μL droplet may continuously alter the local indigo concentration within the laser focal volume. In addition, rapid evaporation during laser exposure caused changes in droplet geometry, focal position, and spectral intensity. Therefore, the concentration-dependent SERS measurements were performed after drying the droplets to provide more stable and comparable acquisition conditions. Future work will evaluate strategies to reduce evaporation, control particle redistribution, and enable more reproducible operando SERS measurements. Therefore, SERS is interpreted as complementary molecular identification, not as an operando validation of the WGFET response.

## 3 Results and Discussion

### 3.1 Morphological Characterizations

The ZnO and ZnO–Ag films uniformly coated the substrates as shown in the optical microscopy images in Figure S3a,b. Figure 2a,b displays the Raman spectra of the ZnO–Ag and ZnO films, respectively. The Raman spectrum of indigo powder and the corresponding blank spectra of the ITO/glass substrates, used as reference controls, are provided in Figure S4a. The Raman spectra obtained from each film reveal significant differences in terms of intensity and spectral complexity (Figure 2). For the ZnO film, the characteristic phonon-related vibrational modes of the wurtzite structure are observed, such as E_2_(high) at ∼437 cm^–1^, assigned to the hexagonal wurtzite crystal structure of ZnO, in addition to the bands at ∼330 cm^–1^ and ∼580 cm^–1^, associated with structural defects and oxygen vacancies. The contribution near 1100 cm^–1^ may contain overlapping signals from the second-order 2LO modes of ZnO^30^ and the Si–O stretching vibrations of the soda–lime–silicate glass substrate,^31^ as indicated by the ITO/glass control spectrum in Figure S4. Therefore, this feature cannot be assigned exclusively to ZnO. For the ZnO–Ag film, besides the characteristic ZnO vibrational modes, additional Raman bands are observed at approximately 671 and 1050 cm^–1^, which are attributed to C–O and C–O–C vibrations of ethyl cellulose (EC), respectively.^32^ Bands located at 1350, 1410, and 1600 cm^–1^ are associated with polyvinylpyrrolidone (PVP), arising from C–N, CH_2_, and lactam C—O vibrational modes, respectively.^33,34^ These features indicate the presence of residual organic components originating from the nanoparticle stabilization and film formulation processes. In the films analyzed, PVP is present as a stabilizing agent of the commercial Ag nanoparticles, while EC was added to the formulation of the ZnO suspensions as a binder, favoring the adhesion and uniformity of the deposited films, as demonstrated in the tape test (Figure S2). The intensification of these organic bands in the ZnO–Ag highlights the role of Ag nanoparticles in Raman amplification (SERS), suggesting the formation of plasmonic hot spots that enhance adjacent molecular vibrations. Thus, the spectrum of ZnO essentially reflects its crystal lattice, that of the ZnO–Ag composite confirms both the maintenance of the semiconductor phase and the optical amplification effect promoted by Ag-NPs. The Raman signatures associated with residual PVP and ethyl cellulose indicate that the UV treatment does not fully remove the organic matrix. Therefore, the sensing interface should be considered as a heterogeneous ZnO/Ag/organic composite surface, rather than as a purely inorganic ZnO/Ag film. This residual organic matrix may partially passivate adsorption sites, modify local wettability, affect ion accumulation, and contribute to variability in the formation of the electrical double layer. Consequently, the electrical response is interpreted as arising from the interaction of indigo with a heterogeneous interfacial environment, rather than exclusively with isolated ZnO or Ag sites. This interfacial heterogeneity may also contribute to the dispersion observed in the extracted electrical parameters and in the apparent Langmuir–Freundlich fitting constant K.

**Figure 2:**
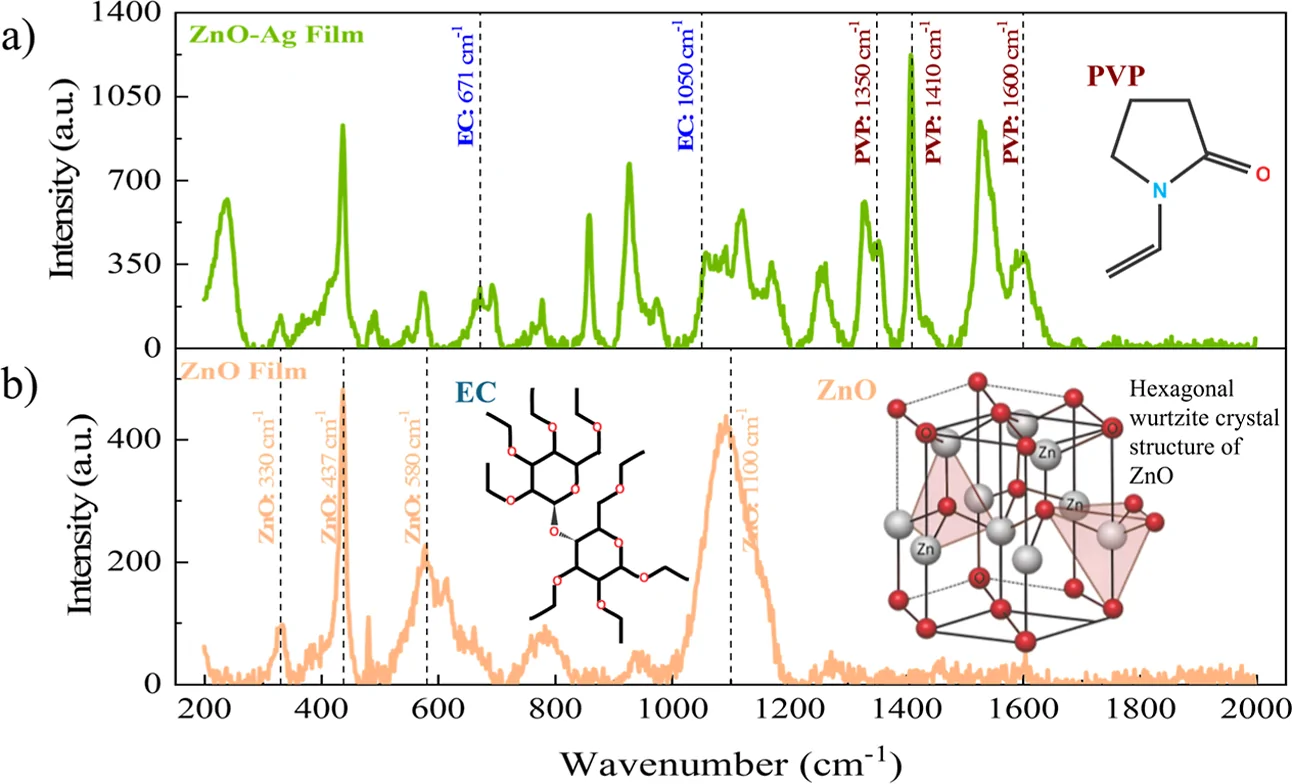
Raman spectra of (a) ZnO–Ag and (b) ZnO films, with dashed lines indicating the characteristic vibrational modes of ZnO, ethyl cellulose (EC), and poly­(vinylpyrrolidone) (PVP). The inset in (a) shows the molecular structure of PVP, while the inset in (b) presents the molecular structure of EC together with the wurtzite crystal structure of ZnO nanoparticles. The spectra were baseline-corrected for clarity.

Figure S5 presents SEM images and the corresponding EDS elemental mapping of the ZnO–Ag film. The SEM micrograph reveals a rough and granular surface morphology on the micrometric scale. The EDS maps show a homogeneous spatial distribution of Zn and O atoms, consistent with a continuous ZnO matrix. In contrast, the Ag signal appears as localized high-intensity regions, forming discrete sub-micrometric clusters with estimated lateral dimensions ranging from approximately 200 to 400 nm.

### 3.2 Electrical Characterization of WGFETs

Due to the presence of active sites such as Zn^2+^ and O^2–^ on the surface of the ZnO-NPs films,^35^ upon addition of water to these surfaces, H_2_O molecules bind to the surface of the ZnO nanoparticles by hydrogen bonding or by chemical adsorption, forming layers of adsorbed water (Figure 3a (II)).^36^ The pH at the point of zero charge (pHpzc) is the solution pH at which the net surface charge is zero under open-circuit equilibrium conditions.^37^ For ZnO, a pHpzc of approximately 8.7 ± 0.2 has been reported.^38^ Therefore, since the pH of ultrapure water (∼7) is below this value, the initially unbiased ZnO surface is expected to be predominantly protonated, forming positively charged surface groups (−OH_2_^+^).^39,40^ During WGFET operation, however, the applied gate potential further modulates the ZnO/electrolyte interfacial charge.^39,40^ This surface loading at the transistor channel acts as an EDL, modulating the carrier density in ZnO and thereby increasing the surface conductivity. Figure 3b shows measurements of electric current as a function of the voltage between the source and drain electrodes for dry ZnO films (blue line, ZnO/Air) and water-covered films (red line, ZnO/Water). An upward shift in the current curve is observed, indicating a reduction in channel resistance promoted by the increase in film conductivity due to the presence of protonated hydroxyl on the film surface. Hysteresis can be seen and is attributed to the hydroxylated ZnO surface due to charge accumulation at the interface.^6^ In addition, because the applied drain–source voltage is swept with time, the channel response can be approximated by an equivalent parallel RC model. In this case, at higher drain–source voltages, the current is predominantly governed by channel conduction, allowing the use of Ohm’s law to estimate effective resistances of 55.56 MΩ and 21.55 MΩ for the dry and water-covered channels at *V*_DS_ = 1.0 V. In contrast, at lower drain–source voltages, the current is mainly associated with interfacial charge accumulation, enabling a capacitive approximation based on *I* = *C­(*d*V/*d*t)*, yielding effective capacitances of 5.0 nF and 89.0 nF for the dry and water-covered channels at *V*_DS_ = 0.5 V, respectively.

**Figure 3:**
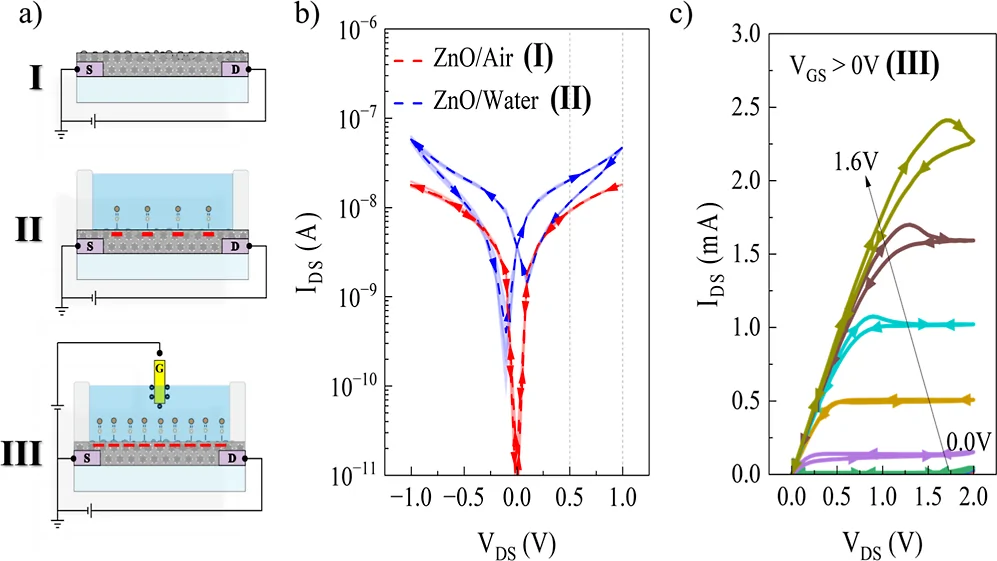
(a) Schematic representation of the device operating states: (I) dry film, (II) film covered with water, and (III) water-gated configuration under applied gate voltage. (b) Current–voltage (*I*–*V*) characteristics of ZnO films measured in air and after water deposition. (c) Output characteristics (*I*_DS_–*V*_DS_) of the WGFET, showing typical n-type behavior under different gate voltages.

Figure 3c shows typical output characteristics of an n-type transistor measured under an applied gate voltage.^6^ It should be noted that, with this configuration, the device can reach currents in the milliampere regime with operating voltages lower than 1.5 V. This is further demonstrated in Figure 4, which shows six cycles of transfer curves for WGFETs using ZnO, Figure 4a, and ZnO–Ag, Figure 4b. For both devices, the six cycles are superimposed, demonstrating moderate and constant hysteresis plus current modulation from ∼10^–7^ A to ∼10^–3^ A, resulting in an ION/IOFF ratio in the range of 10^4^. A slight reduction of *I*_DS_ between 0.0 and 0.3 V is also observed, attributed to the initial reorganization of the water dipoles and the transient adsorption of OH^–^ and H^+^ ions on the semiconductor surface, which reduces the electron density and conductivity of the channel.^41,42^ This initial behavior is followed by an exponential increase in I_DS,_ characteristic of carrier accumulation induced by the formation of the EDL. After the third cycle, the curves stabilize, indicating good reproducibility and the absence of significant degradation for both films. The *I*_GS_ curves as a function of *V*_GS,_ see Figure 4c, show the three distinct conduction regimes in the electrolyte. For *V*_GS_ below approximately 0.3 V, the response is predominantly capacitive, and the measured current is mainly associated with the formation and reorganization of the electrical double layer at the water/semiconductor interface.^41^ Between approximately 0.3 and 0.9 V, the increase in *I*_GS_ suggests the onset of a mixed capacitive–faradaic regime involving interfacial charge-transfer processes.^43^ Above approximately 0.9 V, the sharp increase in *I*_GS_ indicates stronger faradaic contributions, possibly associated with water redox reactions; although the evolution of H_2_ and O_2_ ^44^ was not directly confirmed, it should form the basis of future studies.

**Figure 4:**
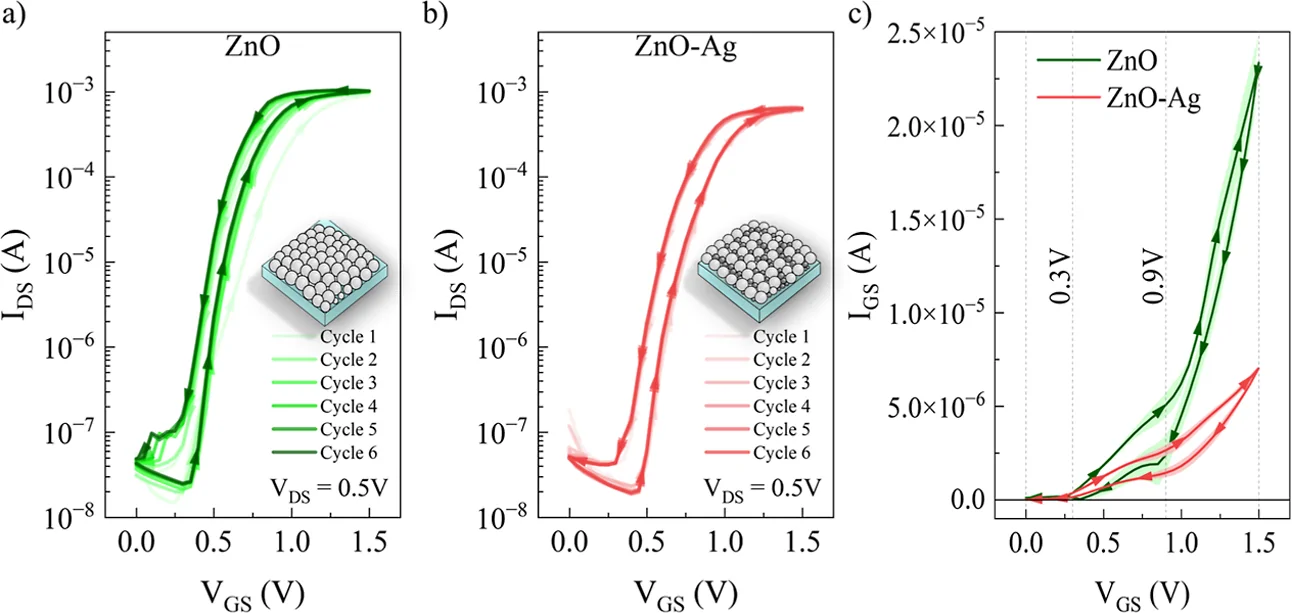
Transfer characteristics recorded over six consecutive scan cycles for WGFETs based on (a) ZnO and (b) ZnO–Ag films, showing good reproducibility and current modulation. (c) Gate leakage current (IGS) as a function of VGS, indicating capacitive, mixed, and faradaic regimes in the electrolyte. Insets schematically depict the ZnO nanoparticle network and Ag nanoparticle incorporation in the ZnO–Ag channel.

Comparison between the devices reveals that the ZnO–Ag transistor exhibits lower gate leakage currents together with more pronounced hysteresis than the pristine ZnO device. The lower gate leakage may contribute to clearer gate modulation; however, the more pronounced hysteresis indicates a stronger contribution of slow interfacial processes at the ZnO–Ag/electrolyte interface compared with pristine ZnO. These processes may include charge trapping, ionic redistribution, adsorption/desorption dynamics, and interfacial polarization effects. Therefore, although the ZnO–Ag interface may enhance the concentration-dependent electrical response, the associated hysteresis should not be interpreted as advantageous for stability or quantitative reproducibility. Instead, it represents an important limitation that must be considered when evaluating the reliability of the sensing response. Device performance was assessed using the on/off ratio, threshold voltage (*V*_th_), transconductance (*g*_m_), subthreshold swing (*SS*), and the mobility–capacitance product (μC), which together describe the switching behavior, sensitivity to gate modulation, turn-on efficiency, and ionic–electronic coupling of the devices. Table 2 summarizes the electrical parameters extracted from the transfer characteristics of five measured devices.

**Table 2:**
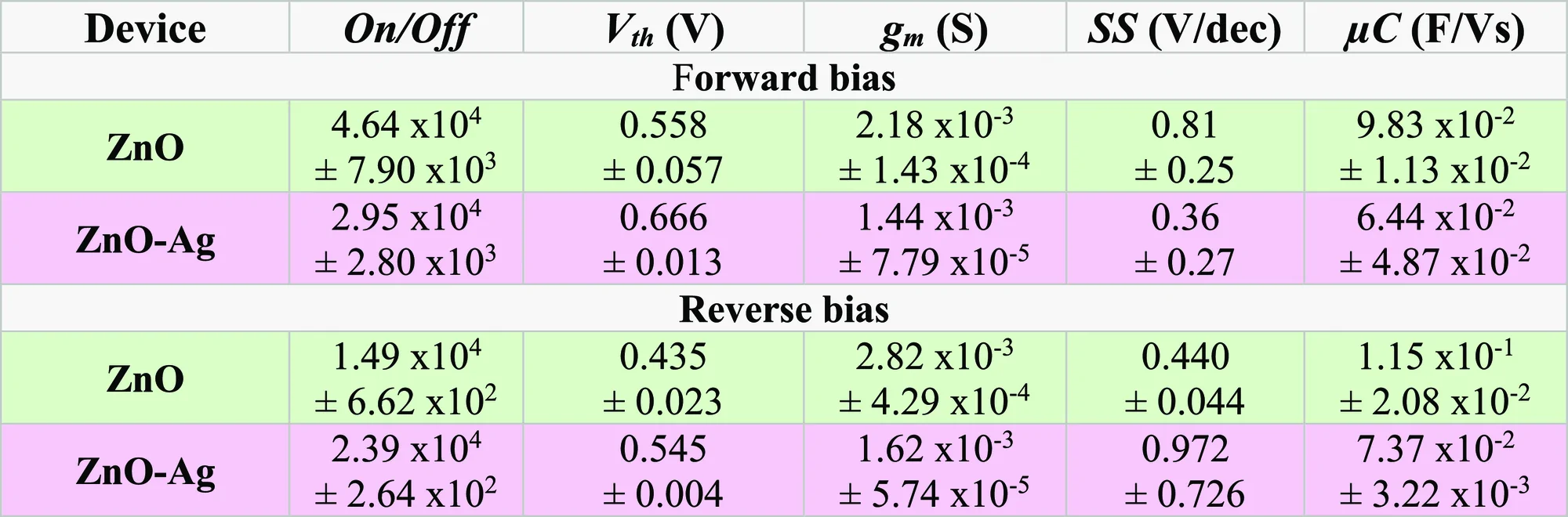
Electrical Parameters Obtained from Five Distinct ZnO and ZnO–Ag Transistors. (*n =* 5)

In addition to controlling the electrostatic modulation mechanism of the channel, the hydroxylated surface of ZnO plays a central role in the interaction with organic analytes in aqueous media. In contact with water, hydration and surface dissociation processes lead to the formation of protonated (−OH_2_^+^) and neutral (−OH) hydroxyl groups, which act as active sites for physical and chemical adsorption.^45^ These surface groups should enable efficient interactions with predominantly neutral organic molecules through hydrogen bonds and short-range interactions, even in the absence of significant electrostatic contributions.^46^ In the case of ZnO–Ag films, the incorporation of silver nanoparticles provides additional interaction sites for indigo while preserving the electrical operation of the transistor, as metallic silver exhibits strong affinity toward organic molecules with π-conjugated systems and heteroatoms, enabling adsorption via Ag–π interactions and electrostatic contributions involving carbonyl groups.^47–49^

### 3.3 Indigo-Induced Modulation in WGFETs

The indigo molecule, in its base form, has carbonyl (C—O) and secondary amine (−NH) functional groups integrated into a conjugated aromatic system,^50^ which can act as hydrogen acceptors and donors. These functionalities allow the formation of donor–acceptor hydrogen bonds with the hydroxyls present on the ZnO surface, especially with the −OH and −OH_2_^+^ groups, favoring molecular anchoring at the ZnO/water interface. Adsorption of dyes on ZnO surfaces is governed by specific local interactions, such as hydrogen bonds and weak coordination interactions involving superficially exposed Zn^2+^ centers, to the detriment of mechanisms based on long-range electrostatic attraction^47–49^

In water-gated transistors, changes in ionic strength, pH, dielectric environment, interfacial capacitance, and electrical double-layer organization may also affect the transfer characteristics. Therefore, the WGFET response is interpreted as a combined effect of indigo presence and electrolyte/interface perturbation rather than as evidence of an exclusively indigo-specific interaction. To support analyte identification, SERS was used as a complementary molecular technique, confirming indigo through its characteristic vibrational bands. Thus, WGFET provides sensitive electrical transduction while SERS provides selective spectroscopic validation. However, the selectivity of the electrical response itself has not yet been fully established. Further control studies using structurally related dyes, charged organic molecules, surfactants, and solutions with controlled pH and ionic strength are required to evaluate cross-sensitivity, quantify selectivity, and distinguish indigo-specific interactions from those of generic electrolyte or interfacial effects.

In WGFET devices, the threshold voltage (*V*_th_) considered in this work is defined operationally as the gate voltage required to reach a predefined drain current level, rather than as an intrinsic transistor parameter; in this sense, the extracted threshold depends on the target current adopted, since different current levels probe distinct conduction regimes and interfacial processes at the semiconductor/electrolyte interface. In this context, the choice of current values is critical to ensure reproducibility, analyte sensitivity, and comparability between ZnO and ZnO–Ag-based devices. The complete cycles for each concentration are shown in Figure S6a,b. Figure S7 shows the variation of the *V*_th_ for 1.0 μA and 10.0 μA, as a function of indigo concentration for devices based on ZnO (Figure S7a,b) and ZnO–Ag (Figure S7c,d), considering the scan direction. In both materials, it is observed that *V*_th_ is sensitive to the presence of the analyte, evidencing the electrostatic modulation of the channel induced by the adsorption of the dye. However, the ZnO–Ag devices exhibit a stronger correlation of *V*_th_ with analyte concentration and a more pronounced difference between forward and reverse scan directions, indicating increased hysteresis.

Figure 5 shows the analysis of the *V*_th_ changes with respect to the reference without analyte (pure water −0.00 M) (Δ*V*_t0_), evidencing the effect of indigo adsorption on the electrical response of the devices. For ZnO films, as shown in Figure 5a,b, the Δ*V*_th0_ shifts occur in a more irregular manner, regardless of the sweep and the target current value. The values of Δ*V*_th0_ obtained do not clearly exceed the reference threshold, defined as three times the standard deviation of the *V*_th_ value measured in water (3σ). In contrast, the ZnO–Ag devices, Figure 5c,d, exhibit well-defined Δ*V*_th0_ shifts relative to the reference, with less data scattering in the reverse scan.

**Figure 5:**
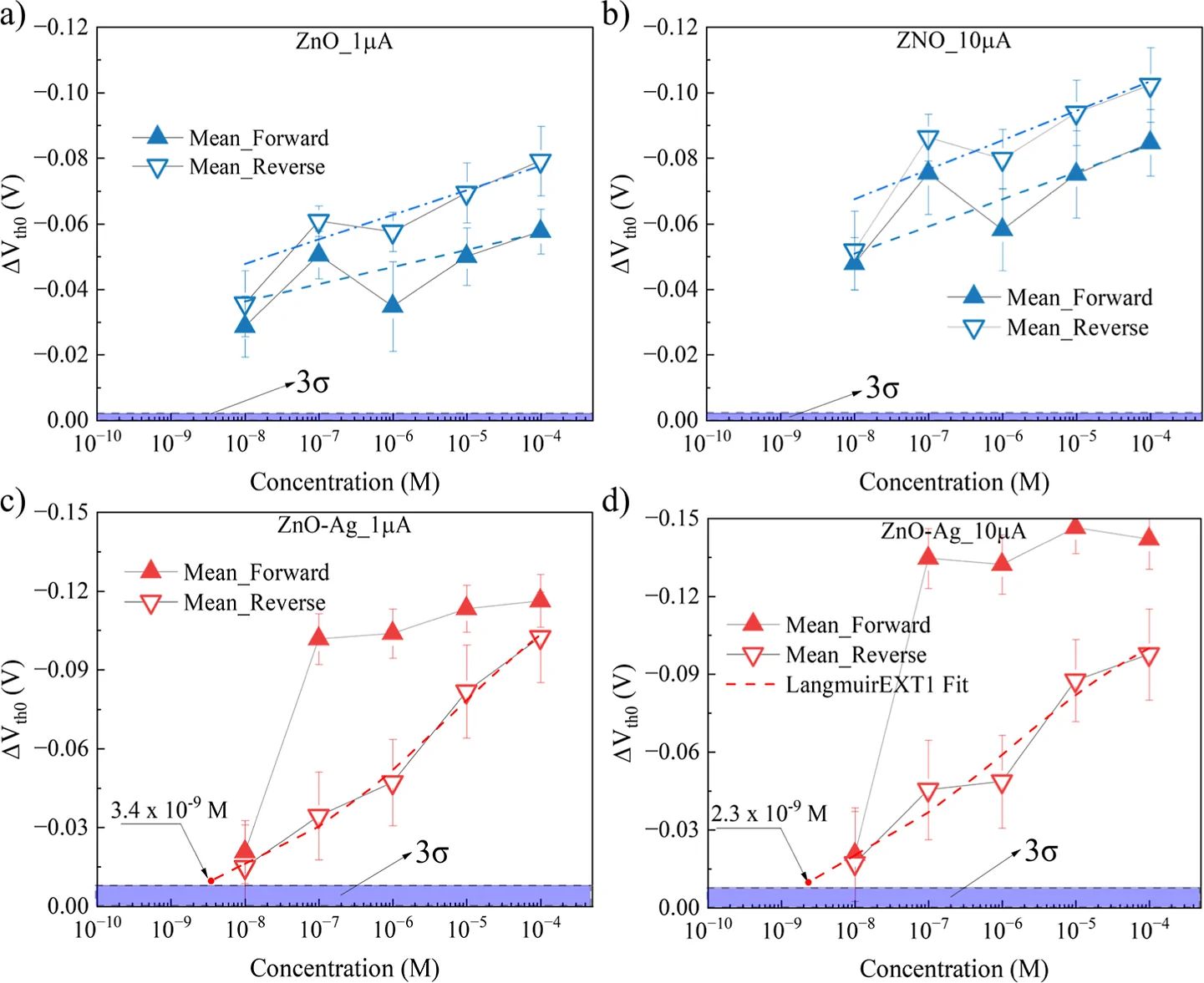
Threshold voltage shifts relative to the reference condition (without analyte, pure water, 0.00 M), expressed as Δ*V*_th0_, as a function of indigo concentration for ZnO (a,b) and ZnO–Ag WGFETs (c,d). Measurements were performed at target drain currents of 1 μA (a,c) and 10 μA (b,d), for forward and reverse gate-voltage sweeps. The shaded region represents the reference threshold defined as three times the standard deviation of the water measurement. Reverse-scan data for ZnO–Ag devices were fitted using the Langmuir–Freundlich adsorption model.

The present WGFET data suggest that exposure to indigo dye modifies the ZnO/Ag/electrolyte interface, producing measurable changes in the transfer characteristics. In water-gated and electrolyte-gated FET sensors, such electrical modulation is commonly associated with changes in the interfacial potential, surface charge distribution, electrical double-layer capacitance, and analyte-induced perturbation of the semiconductor/electrolyte interface.^51,52^ However, in the present work, the specific microscopic contributions of hydrogen bonding, Ag–π interaction, charge transfer, and electrical double-layer perturbation cannot be separated with the current dataset. Therefore, the WGFET response is interpreted as a phenomenological electrical modulation induced by indigo dye at the ZnO/Ag/electrolyte interface, rather than as direct experimental evidence of a specific adsorption or charge-transfer mechanism. Without surface-sensitive measurements, Ag-assisted interfacial interactions cannot be directly demonstrated in the present work, although they remain plausible based on previous reports on Ag-containing SERS interfaces.^53,54^

Consistent with this interpretation, the variation in threshold voltage shift (Δ*V*_th0_) with indigo concentration was analyzed using the Langmuir–Freundlich model,^55^ adapted here to describe the nonlinear electrical response of the WGFET. The use of this model is not intended to establish a specific adsorption mechanism but rather to provide a phenomenological description of the concentration-dependent response, consistent with an apparent heterogeneous interfacial process. In this framework, Δ*V*_th0_ is treated as proportional to the apparent analyte surface coverage, allowing the adsorption isotherm to be expressed as

$${\Delta V}_{th0}\left(C\right)={\Delta V}_{th0,\max}\cdot \frac{{KC}^{n}}{1+KC^{n}}$$

where Δ*V*_th0,max_ represents the maximum threshold voltage shift, K is the apparent adsorption affinity, and n accounts for surface heterogeneity. Fitting the ZnO/Ag 10 μA return-scan data yielded Δ*V*_th0,max_ = −0.122 ± 0.050 V, K = 0.887 ± 0.833, and *n* = 0.674 ± 0.175. The fitted parameters, particularly K, present high relative uncertainty; therefore, K should be interpreted as an apparent fitting parameter rather than an intrinsic adsorption constant. As shown in Figure S5, SEM/EDS mapping reveals a heterogeneous surface morphology with dispersed Ag nanoparticles embedded in the ZnO matrix, which supports the presence of a heterogeneous interface but does not, by itself, confirm a specific adsorption mechanism. Thus, the deviation of n from unity should be viewed only as compatible with a distribution of apparent interfacial responses, possibly associated with ZnO/Ag agglomerations and defective sites, rather than as direct proof of adsorption-energy heterogeneity. Because of the limited number of concentration points, the absence of alternative model comparison, and the lack of residual analysis, the model should not be used as definitive evidence of adsorption heterogeneity or a controlled adsorption mechanism. Consequently, although the calibration trend suggests sensitivity to low indigo concentrations, the extrapolated nanomolar LoD should not be interpreted as a fully reliable or experimentally validated detection limit. It should instead be considered an apparent analytical estimate derived from the calibration procedure using the 3σ criterion. The high standard deviations in the electrical parameters and the uncertainty associated with K limit confidence in extrapolation outside the tested concentration range; therefore, additional measurements at lower concentrations, with more calibration points and independent devices, are required to confirm reliable nanomolar detection.

Thus, the ZnO–Ag device shows a more reproducible electrical response to indigo concentration under the tested conditions, but this should be interpreted as improved empirical sensitivity rather than definitive proof of a controlled adsorption mechanism.

### 3.4 Indigo-Induced Surface-Enhanced Raman Scattering Enhancement

While the WGFET configuration offers high sensitivity to interfacial processes, it is intrinsically non-specific, as any species adsorbing at the semiconductor/electrolyte interface may induce threshold voltage shifts through modulation of the electric double layer. Electrical detection alone therefore cannot unambiguously identify the adsorbed analyte. This limitation is addressed by combining the WGFET response with SERS measurements on the same ZnO–Ag interface, which provides molecular evidence through the vibrational fingerprint of the analyte. Raman spectroscopy enables direct identification of indigo via its characteristic bands, supporting molecular confirmation of the dye. This dual-transduction strategy provides additional selectivity support, since electrical modulation indicates interfacial changes at the ZnO–Ag/electrolyte interface, whereas Raman spectroscopy provides molecular confirmation through the characteristic vibrational bands of indigo. Thus, the platform should be considered a complementary electrical–spectroscopic strategy: the WGFET provides a sensitive real-time response in water, while SERS improves selectivity by confirming the molecular identity of indigo.

After the WGFET measurements, the devices were dried to remove water and form a thin film of indigo on the surface of the films, and Raman spectra as a function of the indigo concentration were then acquired. Figure S4a shows the spectra for the indigo powder and ZnO and ZnO–Ag films. Figure S4b,c shows the Raman/SERS spectra for each substrate containing concentrations between 10^–4^ and 10^–8^ M.

These dried-sample SERS measurements are therefore used as complementary molecular identification of indigo adsorbed on the sensing layer, rather than as an operando validation of the WGFET response. A fixed-concentration SERS measurement carried out in an aqueous medium is included in the Supporting Information, Figure S9, to demonstrate the feasibility of detecting indigo directly in the liquid phase. The liquid-state spectrum exhibited greater temporal variability than the dried-sample measurements. This behavior may be related to the heterogeneous nature of the indigo dispersion at near-neutral pH, since suspended particles and aggregates can move, sediment, or be redistributed within the droplet, producing fluctuations in the number of dye molecules or particles sampled by the laser focal volume. UV–Vis absorption of a CVD-grown indigo dye film, Figure S10, showed a broad visible band centered at approximately 660–670 nm, with absorption extending towards the 532 nm excitation wavelength. Therefore, a pre-resonance Raman contribution cannot be excluded, although it cannot be quantitatively separated from the SERS response under the present experimental conditions.

The SERS results demonstrate molecular identification of indigo dye on the ZnO/Ag sensing layer. Previous SERS and DFT studies of indigo adsorbed on silver nanostructured surfaces have shown that the Raman response of indigo can be modified by its interaction with Ag surfaces.^47^ In addition, ZnO/Ag-based SERS platforms have reported Raman enhancement associated with Ag-related electromagnetic hot spots and interfacial electron-transfer processes between ZnO and Ag.^56^ These literature reports support the plausibility of adsorption-assisted and charge-transfer-assisted SERS enhancement in ZnO/Ag–indigo systems. Nevertheless, because no surface-sensitive or transient measurements were performed here, these mechanisms should be considered plausible interfacial contributions rather than direct experimental conclusions for the present devices.

The choice of bands at 1450 and 1573 cm^–1^ as spectroscopic markers of the dye was based on their vibrational relevance, intensity, and behavior under SERS conditions. The bands near 1450 and 1570 cm^–1^ were selected as spectroscopic markers of indigo based on their vibrational relevance, relative intensity, and behavior under SERS conditions. Although the spectra exhibit a variable background, possibly associated with residual organic species from the AgNP dispersion and spatial heterogeneity in the distribution of AgNPs, hot spots, and indigo molecules, dye identification was based on these characteristic bands rather than on the overall spectral intensity. These bands are attributed to deformation modes of the aromatic skeleton and to conjugated C—C and C—O stretching modes, respectively. Figure 6 shows the Raman spectra obtained for the indigo dye adsorbed on ZnO and ZnO–Ag substrates. In Figure 6a, the ZnO–Ag films exhibit detectable characteristic bands even at low concentrations (10^–8^ M), evidencing a strong SERS amplification effect. This enhancement is discussed as being consistent with possible electromagnetic contributions from Ag nanoparticles and possible chemical/interfacial effects involving the ZnO/Ag surface and indigo molecules.^57^ However, these contributions were not quantitatively separated in the present study. In the ZnO substrate, it was possible to obtain the same dye bands only when the highest concentration (10^–4^ M) was used. The significant increase in peak intensity at 1458 and 1573 cm^–1^ on the ZnO–Ag substrate supports the presence of SERS-active regions associated with Ag nanoparticles without allowing a quantitative separation of plasmonic and chemical/interfacial effects. The main high-wavenumber marker is therefore termed approximately 1570 cm^–1^ throughout the discussion, consistent with the spectrum.

**Figure 6:**
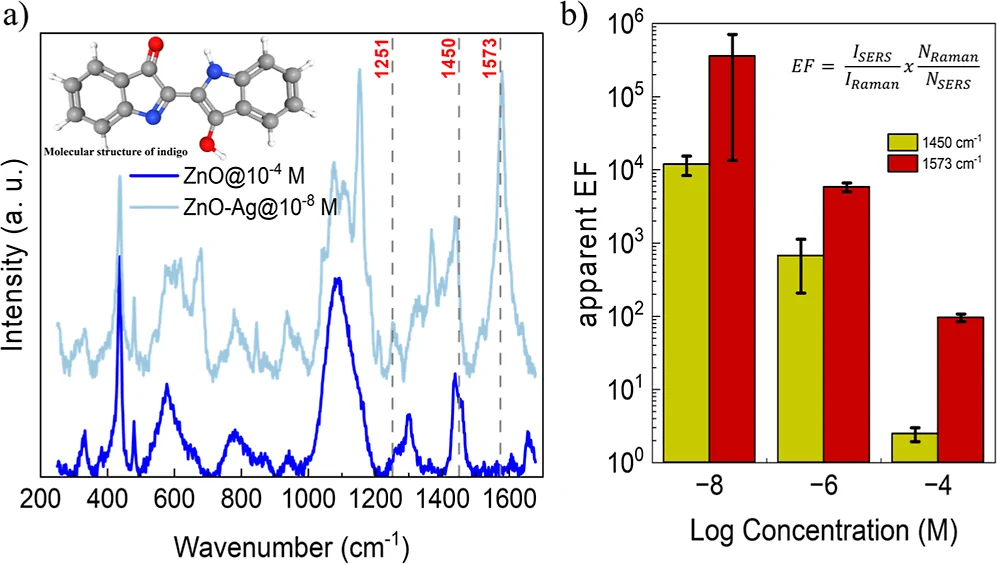
(a) SERS spectra of indigo at concentrations from 10^–4^ to 10^–8^ M, highlighting the characteristic bands at 1451 and 1573 cm^–1^. The inset shows the molecular structure of indigo, indicating the π-conjugated backbone and carbonyl groups relevant for interfacial interactions. (b) Apparent enhancement factors calculated for the 1451 and1573 cm^–1^ vibrational modes on the ZnO–Ag substrate.

The SERS enhancement is discussed as arising from possible combined electromagnetic and chemical and interfacial contributions. However, these mechanisms were not quantitatively separated in the present study. Therefore, the SERS data are used mainly as a complementary molecular confirmation of indigo through its characteristic vibrational bands.

The SERS results are therefore interpreted primarily as molecular confirmation of indigo on the ZnO–Ag sensing layer. The apparent enhancement factors calculated for the main indigo bands provide a comparative estimate of the Raman amplification obtained on the ZnO–Ag substrate under the tested dried-sample conditions, but they should not be interpreted as concentration-dependent intrinsic properties of the substrate.

Table 3 summarizes reported studies on indigo detection, highlighting the substrates used, the measurement conditions (solid state or aqueous medium), and the mechanisms responsible for SERS intensification. Indigo carmine is a sulfonated derivative of indigo, soluble in water, containing two sulfonate groups (−SO_3_^–^) and can exist in different protonation states depending on pH. Figure S8 presents a comparison between the molecular structures of indigo and indigo carmine. In aqueous media, indigo carmine can be reliably detected with LoD on the order of 5 × 10^–9^ mol L^–1^ when adsorbed on Ag-based substrates due to the combination of electromagnetic and chemical intensification mechanisms. In contrast, indigo in its base form (indigotin), due to its low solubility, is mostly detected in adsorbed or solid-state configurations, in which the SERS spectra are dominated by bands close to 1450 and 1573 cm^–1^. In this work, the SERS LoD was defined as the lowest indigo concentration yielding a signal-to-noise ratio higher than 3, corresponding to 1 × 10^–8^ mol L^–1^. For the WGFET response, an apparent electrical LoD of 4 × 10^–9^ mol L^–1^ was estimated from the Langmuir–Freundlich fit using the 3σ criterion and should therefore be interpreted as an extrapolated analytical estimate under the tested conditions. Overall, the comparison indicates that the present study achieves a performance compatible with the detection of indigo-based dyes under aqueous conditions. This performance is attributed to the combined electrical sensitivity of the ZnO–Ag/electrolyte interface and the SERS activity provided by Ag nanoparticles incorporated into the ZnO matrix. The Ag-containing interface may favor local field enhancement and molecular adsorption; however, the individual contributions of adsorption-site density, charge transfer, and interfacial electronic coupling were not quantitatively separated in this work.

**Table 3:** Publications Related to Indigo Detection in the Base Form via the SERS Technique

Technique	substrate utilized	type of indigo	sample/context	peaks observed (cm^–1^)	LOD (mol L^–1^)	year/ref
SERS	ZnO nanoplates decorated with Ag NPs	indigo carmine	drop-cast and adsorbed	1254, 1352, 1585	5 × 10^–9^	2025 ^51^
multi-step SERS	colloid Ag (citrate)	indigo base (in mixture)	indigo + yellow pigment blends	532–1582	∼10^–7^	2016 ^58^
conventional SERS	Ag NPs colloidal (citrate)	indigo base	solid indigo and aqueous solutions	545, 598,1225, 1311, 1573	∼10^–6^	2017 [48]
SERS + DFT (Ag_16_)	Ag nanofilm (PLD) + Ag_16_ model	indigo base	indigo adsorbed on polished substrate	546, 598, 1016, 1312, 1368, 1576	∼10^–7^	2018 ^47^
SERS in Gel	AgNPs paste on polymeric gel	indigo base	fabrics and mock paintings	544, 597, 1250, 1573	∼10^–8^	2023 ^59^
WGFET + SERS	ZnO/Ag film	indigo base	solid indigo and aqueous solutions	1450, 1573	SERS: 1 × 10^–8^; WGFET: apparent 4 × 10^–9^	This Work

## 4 Conclusion

ZnO- and Ag-based nanocomposites were employed as active layers for the detection of indigo dye in its base form through the integration of WGFET electrical transduction and SERS optical analysis. The incorporation of Ag nanoparticles into ZnO films produced measurable changes in leakage current, hysteresis, and threshold voltage response, indicating that the ZnO/Ag/electrolyte interface is highly sensitive to indigo exposure. The Langmuir–Freundlich model was used as a phenomenological description of the nonlinear concentration-dependent response; however, the limited number of concentration points and the uncertainty of the fitted parameters mean that the model should not be interpreted as definitive proof of adsorption heterogeneity or controlled adsorption. The extrapolated electrical LoD should therefore be considered an apparent analytical estimate under the tested conditions. SERS measurements provided molecular confirmation of indigo through its characteristic vibrational bands, complementing the sensitive but non-specific WGFET response. Overall, the WGFET/SERS platform is presented as a proof-of-concept integrated sensing strategy for local screening and molecular confirmation of indigo in water, while further studies involving selectivity assays, complex environmental matrices, XPS, electrochemical impedance spectroscopy, transient measurements, and adsorption/desorption kinetics are required for full mechanistic validation and practical deployment.

## Supplementary Material



## Notes

Photographs of aqueous indigo dispersions; tape adhesion tests of ZnO films; optical microscopy images of ZnO and ZnO–Ag films; Raman spectra and reference controls; SEM images and EDS elemental mapping; transistor transfer characteristics and threshold-voltage analysis; molecular structures of indigo and indigo carmine; liquid-droplet SERS spectrum; and UV–Vis absorption spectrum of an indigo film (PDF)
