# Supplementary material for: Zinc Oxide/Ag Water-Gated Field-Effect Transistor Coupled with Surface-Enhanced Raman Scattering for Indigo Dye Detection
Source: ACS Omega. 2026 Sep 11;11(37):55442–53. doi: 10.1021/acsomega.6c04192 (PMC13613954; doi:10.1021/acsomega.6c04192)
Supplement: Supplementary file 1 [file ao6c04192_si_001.pdf]

Zinc Oxide/Ag Water-Gated Field-Effect Transistor Coupled with Surface-Enhanced Raman Scattering for Indigo Dye Detection

Rogério Miranda Morais[1, 2]*, Theodoros Serghiou[1], Neri Alves[2], Carlos José Leopoldo Constantino[2], Jeff Kettle[1]

[1]James Watt School of Engineering, University of Glasgow, Glasgow, Scotland G12 8QQ, UK
[2]Department of Physics, School of Technology and Applied Sciences, São Paulo State University (UNESP), Presidente Prudente, SP 19060-900, Brazil

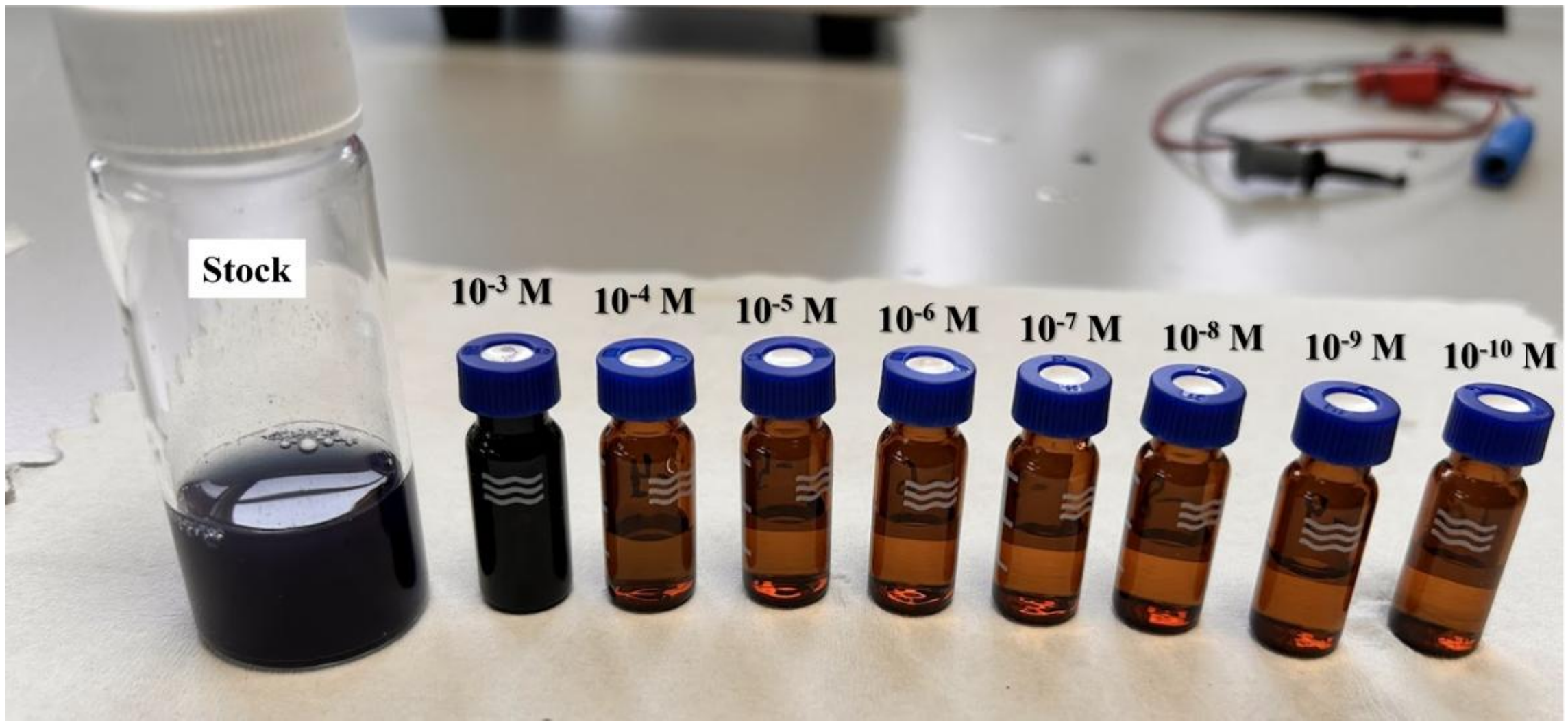

Figure S1. Photographs of aqueous indigo dispersions at concentrations ranging from 10^{-10} to 10^{-3} M after ultrasonic agitation, illustrating the visual appearance and stability of the dispersions prior to device measurements.

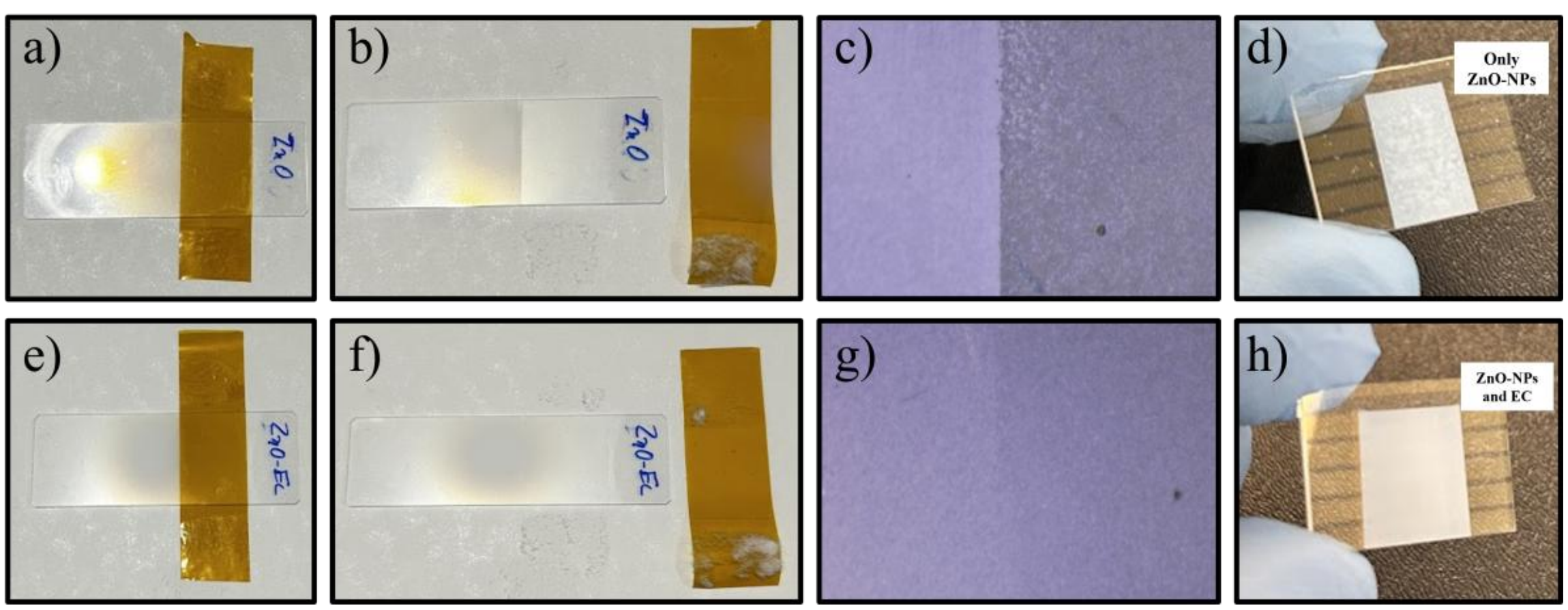

Figure S2. Tape adhesion test of spray-coated ZnO-NP films deposited after thermal treatment without EC (a–d) and with ethyl cellulose (EC) (e–h). (a,e) Films before tape removal; (b,f) films after tape removal; (c,g) optical micrographs of the film edge; and (d,h) ZnO-NP-based films on the patterned substrates after the adhesion test.

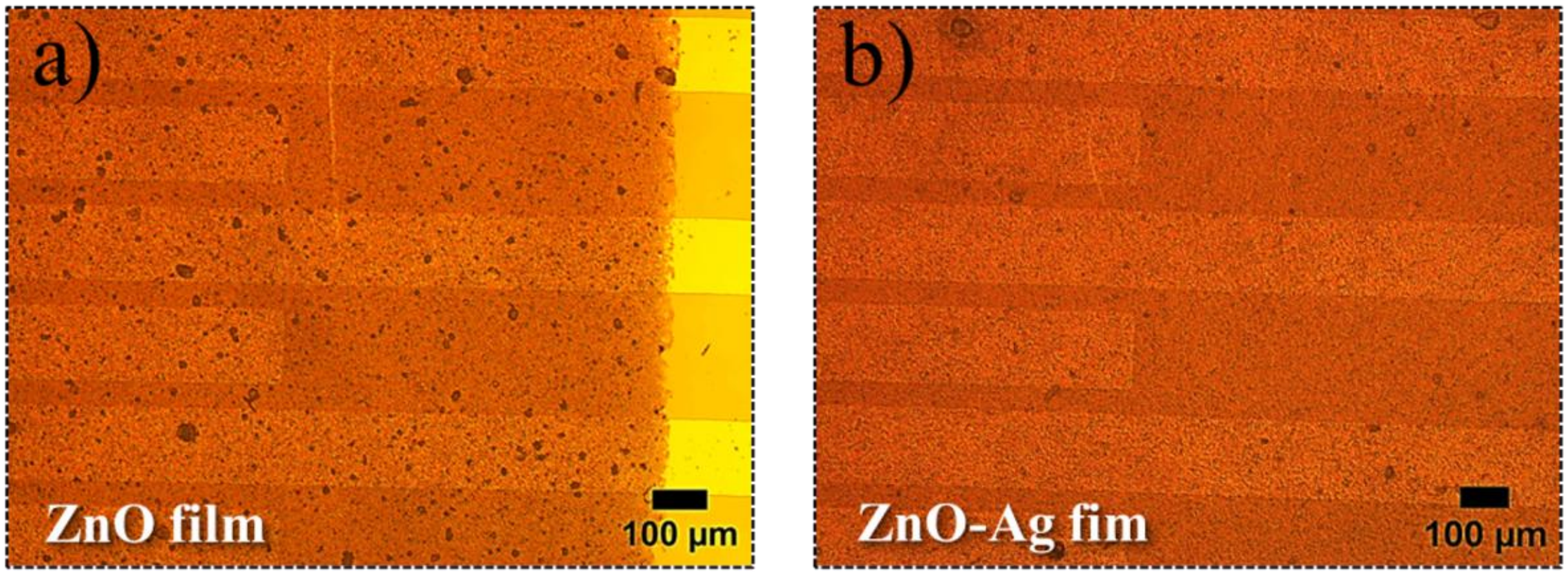

Figure S3. Optical microscopy images of (a) ZnO and (b) ZnO–Ag films deposited by spray coating onto ITO/glass interdigitated electrodes (IDE). Both films exhibit continuous coverage over the substrate at the micrometric scale.

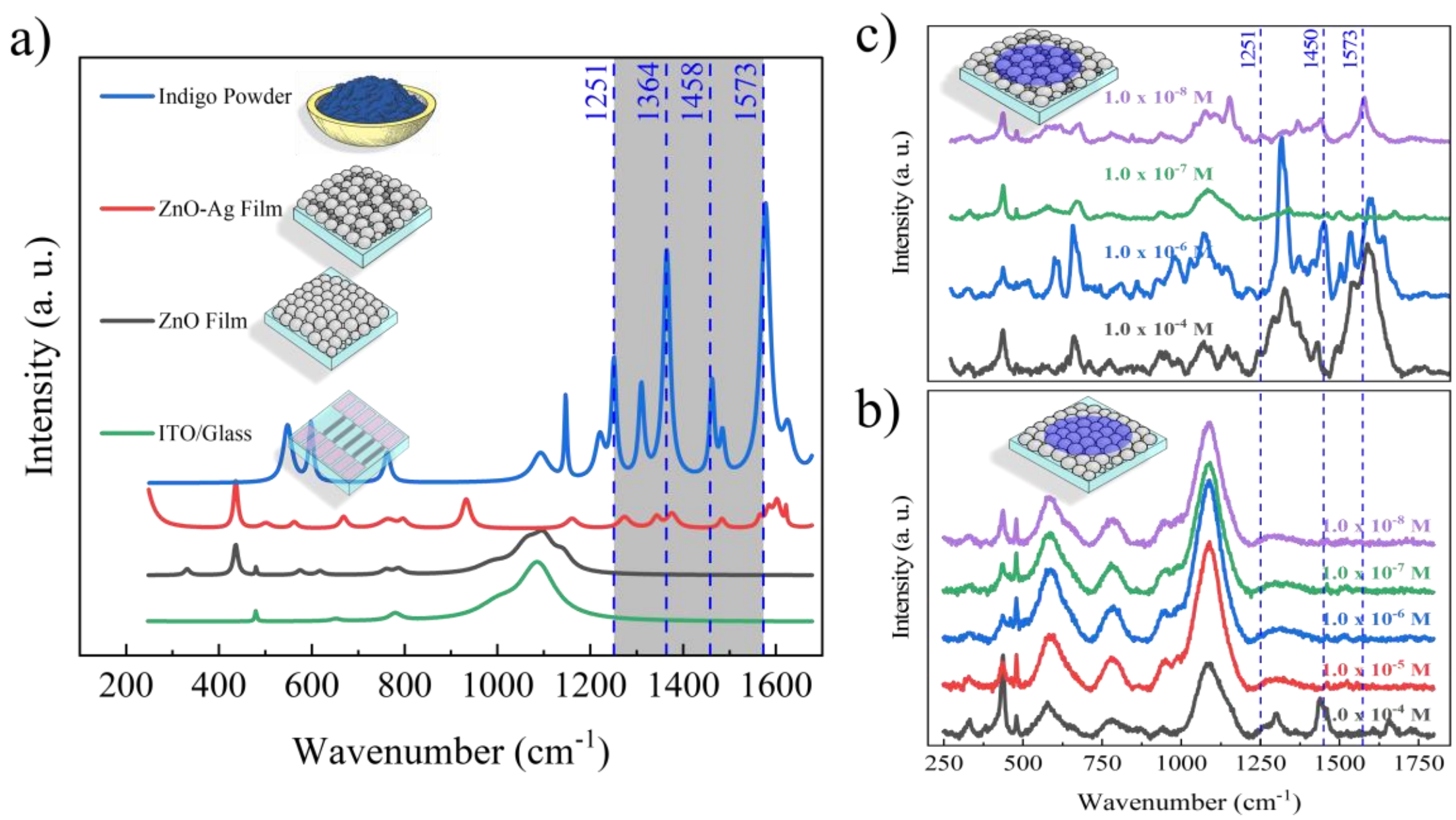

Figure S4. a) The Raman spectrum of the indigo powder and the corresponding blank spectra of the ZnO-Ag film, ZnO film, and ITO/glass substrates, used as reference controls highlighting the characteristic vibrational modes of the dye. (b) Raman spectra of indigo adsorbed on ZnO films and (c) SERS spectra of indigo adsorbed on ZnO-Ag films for concentrations ranging from 10^{-8} to 10^{-4} M. The spectra were baseline-corrected for clarity.

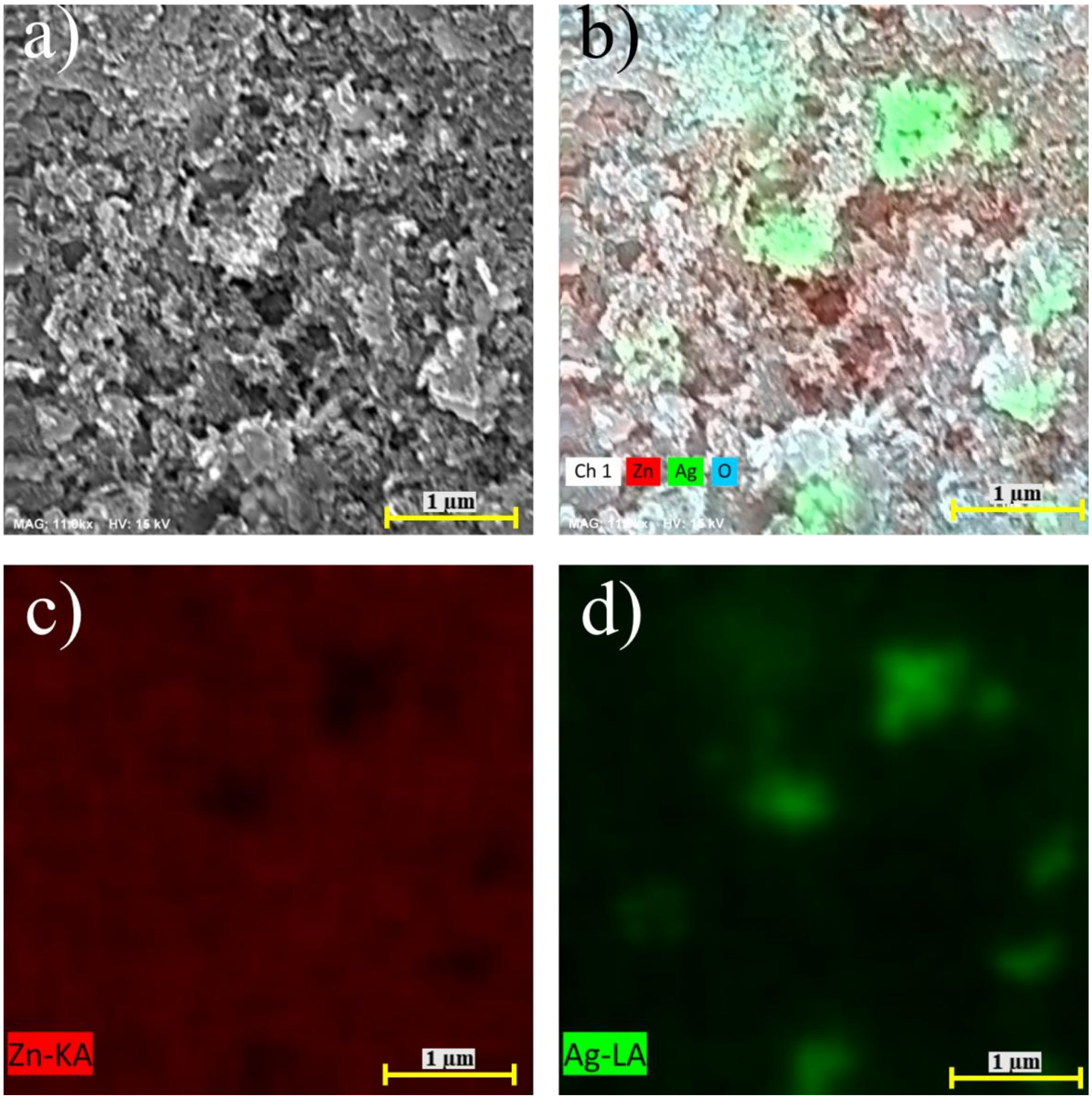

Figure S5. Morphological and compositional characterization of the ZnO-Ag surface used in the sensors. (a) Scanning electron microscopy (SEM) image of the surface of the ZnO-Ag film. (b) Composite elemental distribution map obtained by EDS, simultaneously showing the signs of Zn (red), Ag (green) and O (cyan). (c) Individual elemental mapping of Zn-KA (red), highlighting the continuous distribution of zinc oxide on the surface. (d) Individual elemental mapping of Ag-LA (green), indicating aggregate regions of silver nanoparticles on the ZnO matrix.

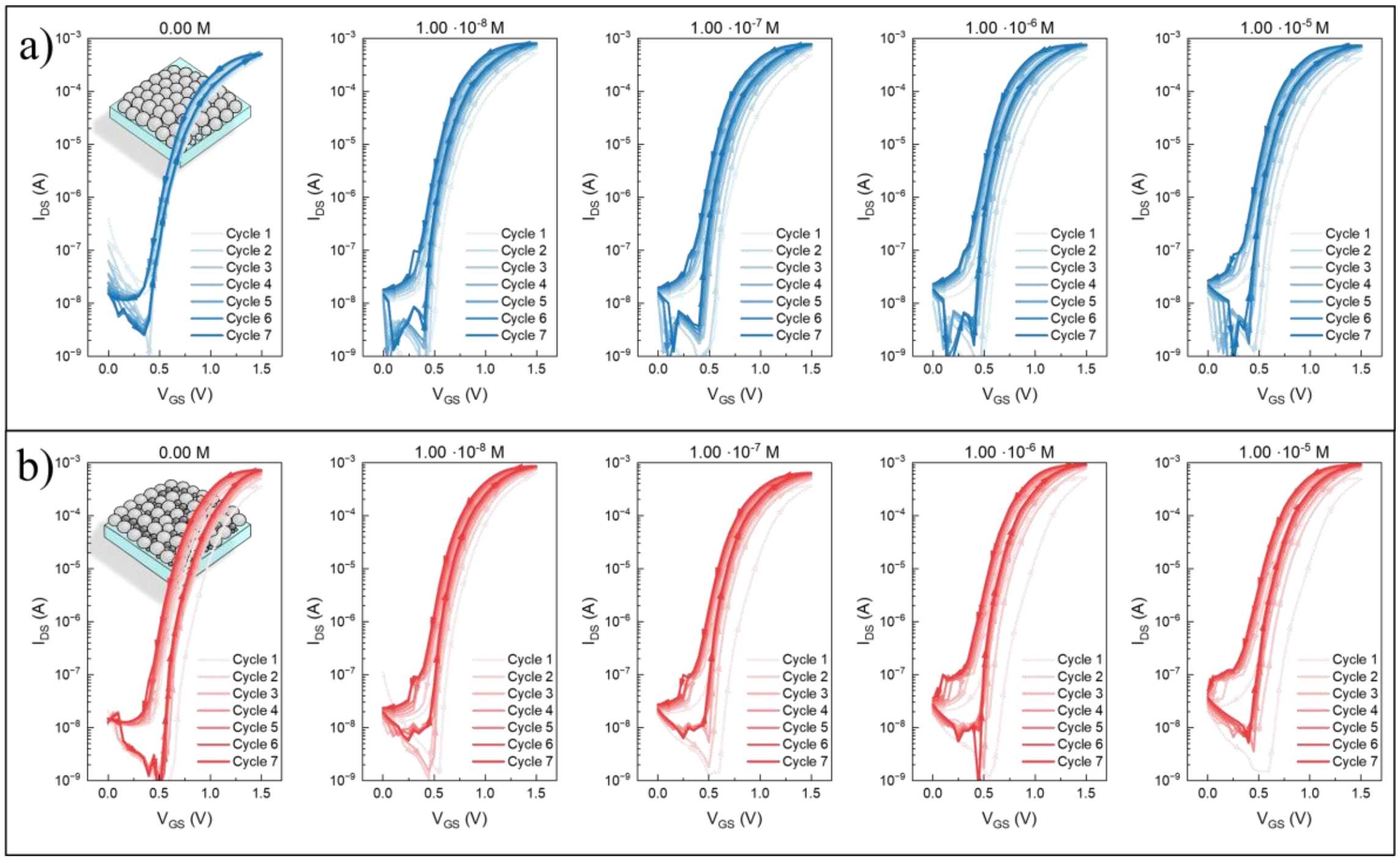

Figure S6. Complete transfer characteristics (I_{DS}–V_{GS}) for all indigo concentrations measured in WGFETs based on (a) ZnO and (b) ZnO-Ag films, including forward and reverse gate-voltage sweeps over six consecutive cycles.

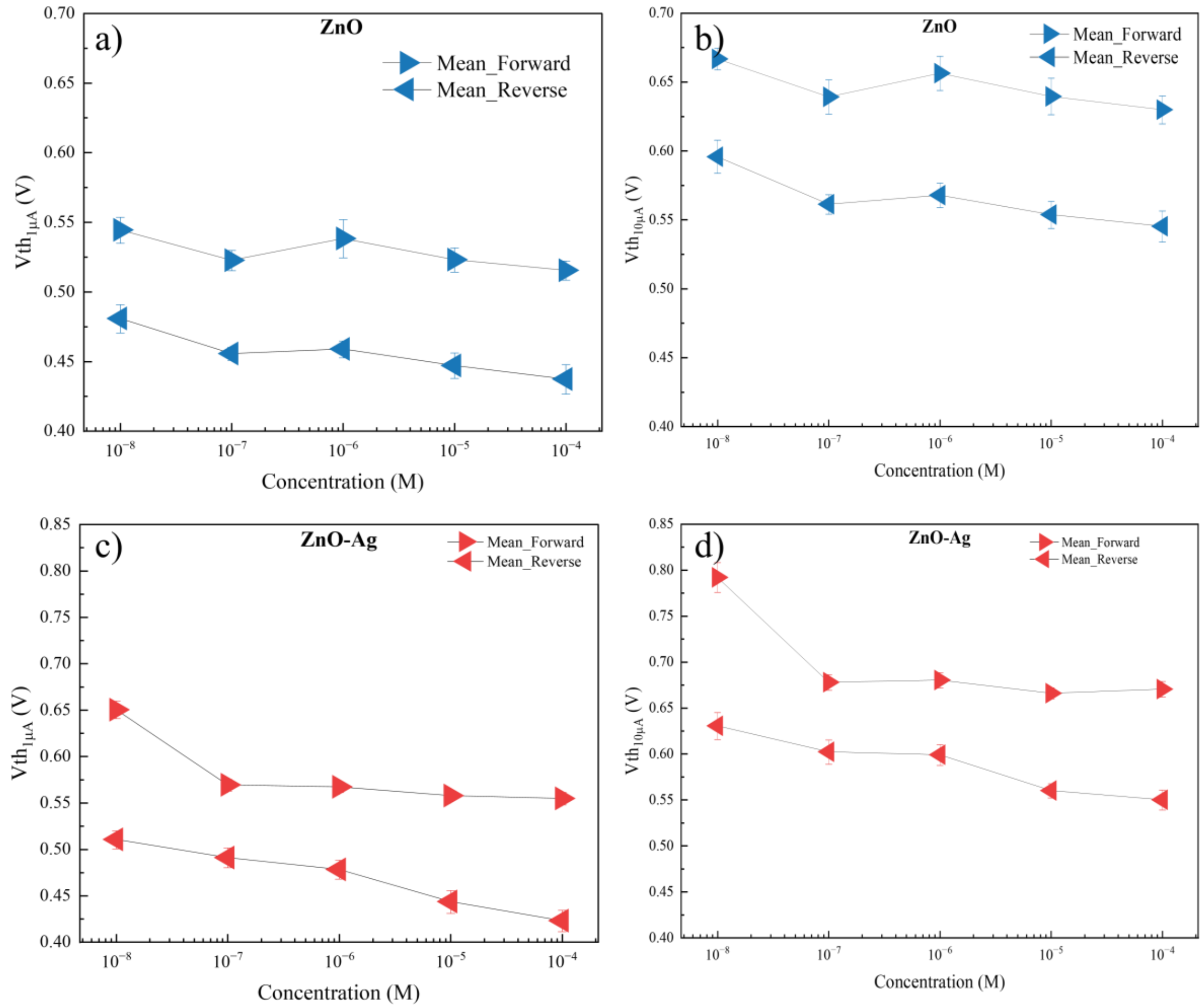

Figure S7. Threshold voltage (Vt) as a function of indigo concentration extracted at target drain currents of (a) 1 µA and (b) 10 µA for ZnO based devices, and (c) 1 µA and (d) 10 µA for ZnO-Ag based devices, considering both forward and reverse sweep directions.

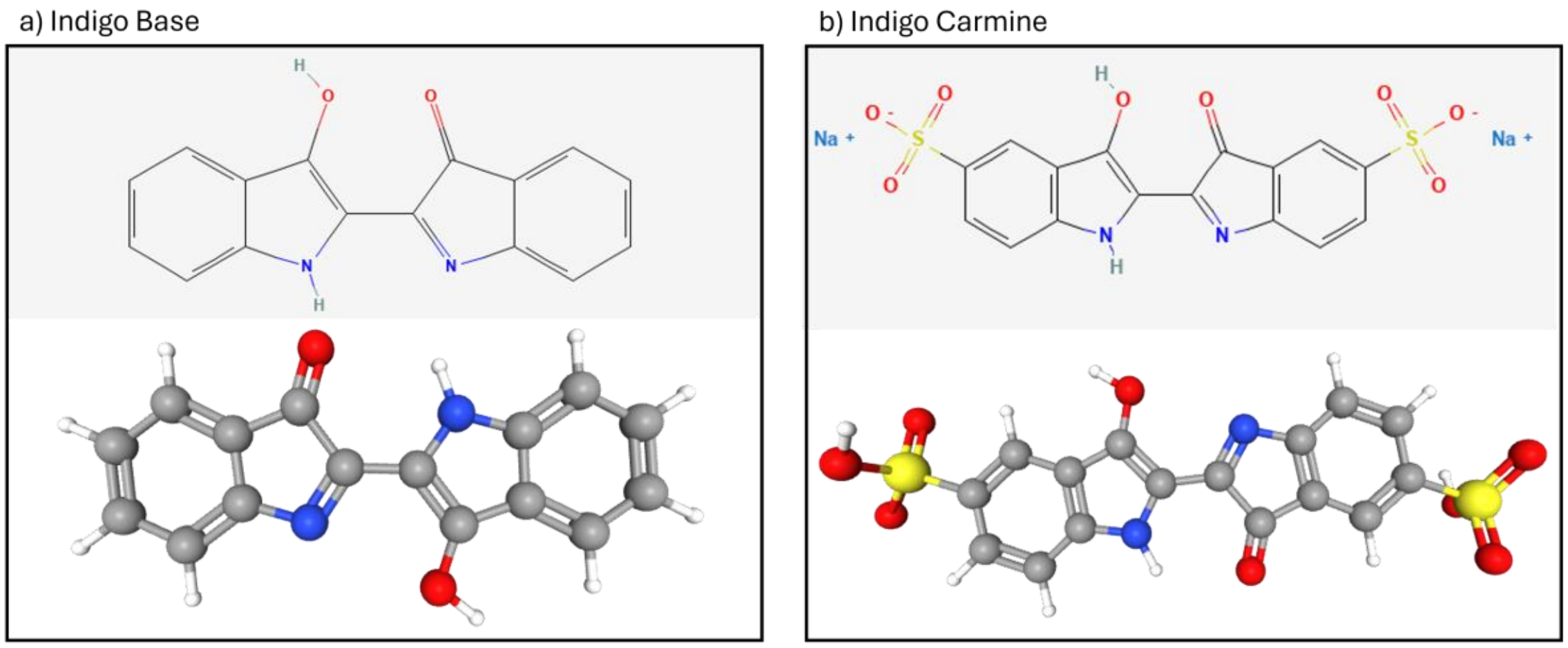

Figure S8. Comparison between the molecular structures of indigo (a) and indigo carmine (b), shown in both 2D (top) and 3D (bottom) representations. Indigo exhibits a planar π-conjugated backbone with carbonyl (C=O) and amine (–NH–) groups, favoring intermolecular hydrogen bonding and limited solubility in water. In contrast, indigo carmine contains two sulfonate groups ($–SO_3^−$), typically balanced by Na^+ counterions, which introduce negative charges, increase hydrophilicity, and enhance aqueous solubility. These structural modifications also influence intermolecular interactions and adsorption behavior at interfaces.

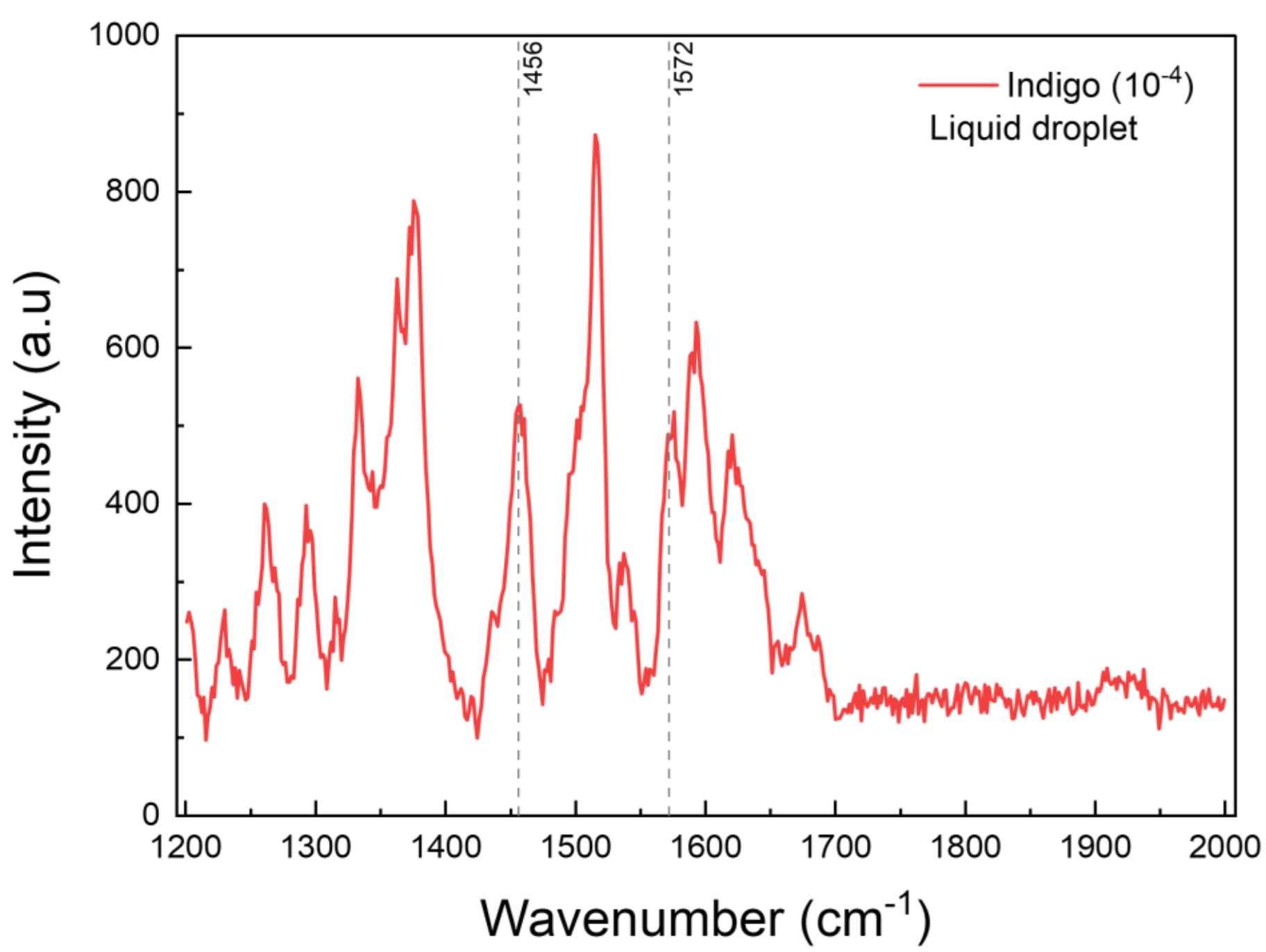

Figure S9. Liquid-droplet SERS spectrum of indigo acquired before droplet drying.

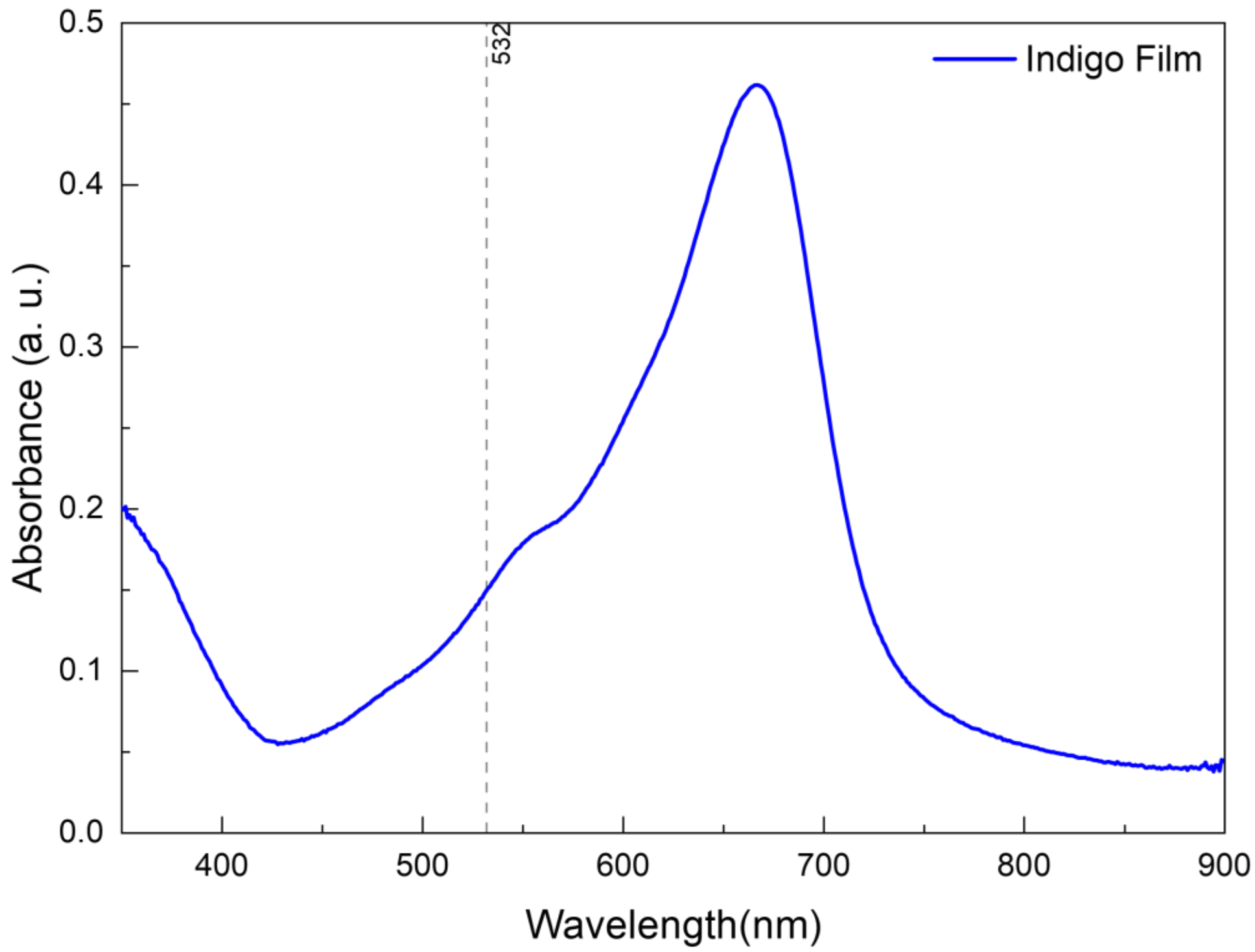

Figure S10 - UV–Vis absorption spectrum of the CVD-grown indigo film. The dashed vertical line marks the 532 nm excitation wavelength used for Raman/SERS measurements
